# Anatomical and morphological predictors of pulmonary arteriovenous malformation recanalisation following embolisation: a retrospective cohort study

**DOI:** 10.1186/s42155-026-00747-y

**Published:** 2026-08-05

**Authors:** Chai Jin Lim, Ahmad Barotchi, Yousef Shahin

**Affiliations:** 1 Medical School, University of Sheffield, Sheffield, UK; 2 Faculty of Health, University of Sheffield, Sheffield, UK; 3 Sheffield Vascular Institute, Sheffield Teaching Hospitals, Sheffield, UK

**Keywords:** Pulmonary arteriovenous malformation, PAVM, Embolisation, Recanalisation, Interventional radiology

## Abstract

**Purpose:**

Embolisation represents the primary therapeutic intervention for pulmonary arteriovenous malformations (PAVMs); however, post-procedural recanalisation remains a recognised complication. This study aimed to identify anatomical and morphological factors associated with PAVM recanalisation, while also describing embolisation modalities used.

**Methods:**

A retrospective, observational analysis was conducted on 40 patients with PAVMs embolised. Demographic variables, angiographic measurements (supplying artery, vein, sac diameters), embolisation modalities including coils, Amplatzer vascular plugs (AVPs), microvascular plugs (MVPs), or combinations, and the presence of recanalisation (defined as < 70% reduction in linear sac diameter on follow-up CT angiography) were collected. Logistic regression analysis was performed.

**Results:**

A total of 109 pulmonary arteriovenous malformations (PAVMs) were embolised across 40 patients, with follow-up imaging available for 86 PAVMs. Larger feeding artery diameter (OR 1.410, *p* = 0.008) and complex PAVM morphology (OR 5.464, *p* = 0.002) were significantly associated with recanalisation on univariate analysis. Male sex and greater sac diameter were also associated on univariate analysis but did not remain significant in the multivariable model. On multivariable logistic regression, complex PAVM morphology (OR 4.00, 95% CI 1.27–12.66, *p* = 0.018) and larger feeding artery diameter (OR 1.29 per mm, 95% CI 1.01–1.65, *p* = 0.044) were independently associated with recanalisation. Although not reaching statistical significance, there was a trend toward higher recanalisation rates in lesions treated with coil embolisation (OR 1.926, *p* = 0.166). Embolisation with vascular plugs was associated with less risk of recanalisation but was insignificant (AVP [OR 0.782, *p* = 0.654], MVP [OR 0.342, *p* = 0.078]).

**Conclusion:**

Larger feeding artery diameter and complex PAVM morphology were independently associated with recanalisation on multivariable analysis. Male sex showed a univariable association that did not persist after adjustment and should be considered exploratory. Device type showed trends but was not a primary determinant. Larger, prospective studies are needed to validate these findings and optimise embolisation strategies.

## Introduction

Pulmonary arteriovenous malformations (PAVMs) are rare vascular abnormalities in which pulmonary arteries and veins are directly connected, resulting in an abnormal right-to-left shunt in the lungs [1]. The definitive treatment for PAVMs is percutaneous transcatheter embolisation which has largely supplanted surgical resection and is now regarded as the gold-standard intervention for PAVMs [2].

Despite advances in embolisation techniques and devices, recanalisation remains a major challenge in terms of post-embolisation complications [2]. Recanalisation may result in persistent right-to-left shunting, necessitating repeat procedures and exposing patients to cumulative procedural risks. Reported rates of recanalisation vary widely. Multiple factors have been postulated to influence recanalisation risk, such as age, sex, presence of hereditary haemorrhagic telangiectasia (HHT), type of PAVM (simple or complex), size of feeding artery, size of sac, and embolisation agents [3]. However, much of the existing evidence derives from relatively small, heterogeneous cohorts, and findings remain inconsistent across studies.

A recent meta-analysis by Shahin et al. found that while embolisation using vascular plugs was associated with higher immediate technical success rates, there was no significant difference in recanalisation rates between different embolisation agents [4]. This suggests that factors other than the choice of embolic material may play a more significant role in determining recanalisation risk.

Post-embolisation PAVM recanalisation is clinically important, yet few studies have systematically evaluated lesion-level anatomical and morphological predictors. We aimed to identify risk factors for recanalisation in a tertiary centre cohort, hypothesising that larger feeding artery diameter, larger sac size, complex PAVM morphology, and embolic device type increase the risk.

## Materials and methods

### Study design and patient selection

We conducted a retrospective, observational study of patients undergoing PAVM embolisation at a tertiary referral centre between 2008 and 2021. Patients were identified via the institution's electronic patient record (EPR) system. Patients with radiologically confirmed PAVMs, who underwent transcatheter embolisation, and had at least one follow-up imaging assessment were included in this study. Patients with incomplete clinical data or missing imaging were excluded. The requirement for informed consent was waived due to the retrospective nature of the study.

### Data collection

Demographic, clinical, and procedural data were systematically extracted from the EPR. Demographic variables, angiographic measurements (feeding artery and venous sac diameters), embolisation modalities including coils, Amplatzer vascular plugs (AVPs), microvascular plugs (MVPs), or combinations, and the presence of recanalisation were collected. Follow-up computed tomography pulmonary angiography (CTPA) was reviewed to assess PAVM morphology and detect recanalisation. The primary outcome was recanalisation, defined as less than 70% reduction in linear sac diameter compared with pre-embolisation measurements, a threshold previously used to indicate inadequate occlusion and persistent or recurrent shunting following embolisation [5]. This threshold represents a pragmatic criterion derived from prior literature rather than a universally standardised definition, and measurement variability between studies should be noted when interpreting these findings. Imaging measurement and analysis were performed independently by CJL and YS; any discrepancies were resolved by consensus.

### Statistical analysis

All statistical analyses were performed using Stata 18 (StataCorp LLC, College Station, TX, USA). Continuous variables were expressed as mean ± standard deviation or median with interquartile range, depending on data distribution, and categorical variables as counts and percentages. Univariate logistic regression was performed to evaluate potential predictors of recanalisation, including demographic factors, anatomical measurements, PAVM complexity, and embolic agent used [4]. Variables with *p* < 0.10 on univariate analysis and strong biological plausibility were considered for multivariable logistic regression. Neither feeding artery diameter nor complex PAVM morphology was included as predictors of recanalisation; sac diameter was excluded due to correlation with feeding artery size. Cluster-robust standard errors were used to account for the inclusion of multiple PAVMs per patient. Statistical significance was set at *p* < 0.05. Odds ratios (ORs) with 95% confidence intervals (CIs) and *p*-values were reported.

## Results

A total of 40 patients with 109 PAVMs underwent embolisation, with follow-up imaging available for 86 (78.9%) PAVMs. Baseline patient and lesion characteristics are summarised in Table 1. Median follow-up was 12 months. Recanalisation occurred in 27 (31.4%) PAVMs. Of those, 14 were embolised with coils, 7 with AVPs, and 6 with MVPs.

**Table 1:** Baseline characteristics of embolised PAVMs. Percentages are calculated at the PAVM level

Variable	Value
Age, Mean (SD)	53 (17)
Male sex, n (%)^a^	20 (23%)
Presence of HHT, n (%)	68 (79%)
Complex PAVM, n (%)	19 (22%)
Artery Diameter (mm), median (IQR)	3.7 (2.6–5.0)
Sac Diameter (mm), median (IQR)	12.9 (10.1–19.6)
Coils Only, n (%)	31 (36%)
AVP only, n (%)	21 (24%)
MVP only, n (%)	23 (27%)
Coils and AVP, n (%)	5 (6%)
Coils and MVP, n (%)	4 (5%)

Data presented as number (%) unless stated otherwise

*SD *Standard Deviation*, HHT *Hereditary Haemorrhagic Telangiectasia*, PAVM *Pulmonary Arteriovenous Malformation*, IQR *Interquartile Range*, AVP *Amplatzer Vascular Plug, *MVP *Micro Vascular Plug

a Refers to number of PAVMs from male patients (20 out of 86 PAVMs with follow-up imaging, 23%)

On univariate logistic regression analysis (Table 2), male sex, larger feeding artery and venous sac diameters, and complex morphology were significantly associated with an increased risk of recanalisation. The presence of HHT and patient age was not significantly associated with outcome. On multivariable logistic regression, complex PAVM morphology (OR 4.00, 95% CI 1.27–12.66, *p* = 0.018) and larger feeding artery diameter (OR 1.29 per mm increase, 95% CI 1.01–1.65, *p* = 0.044) were independently associated with recanalisation. Male sex did not retain significance in the multivariable model and should be interpreted with caution given the methodological limitations of lesion-level analysis for patient-level variables.

**Table 2:** Univariate logistic regression analysis of risk factors for PAVM recanalisation

Variable	Odds Ratio	Standard Error	95% CI	*P* value
Age	0.996	0.014	0.970–1.024	0.790
Male sex^*^	3.523	1.868	1.236–9.961	0.018
Presence of HHT	0.388	0.211	0.134–1.125	0.081
Complex PAVM^†^	5.464	3.034	1.840–16.224	0.002
Feeding Artery Diameter (mm)^†^	1.410	0.182	1.096–1.815	0.008
Sac Diameter (mm)^†^	1.052	0.023	1.007–1.098	0.022
Coils only	1.926	0.912	0.761–4.871	0.166
AVP only	0.782	0.430	0.266–2.296	0.654
MVP only	0.342	0.208	0.104–1.127	0.078
Coils and AVP	3.360	3.172	0.528–21.375	0.199
Coils and MVP	2.154	2.214	0.287–16.146	0.455

^*^ Statistically significant on univariate analysis only; did not persist in multivariable model

† Statistically significant on univariate analysis

**Fig. 1:**
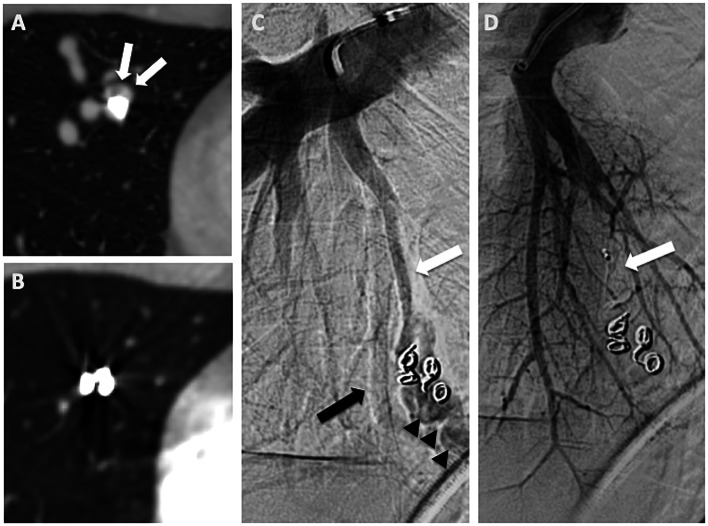
Right middle lobe pulmonary AVM in a 57-year-old female patient with hereditary haemorrhagic telangiectasia which was treated 5 years earlier with coil embolisation. Axial follow-up CT slice (**A**) showing recanalisation after coil embolisation (white arrows). Axial CT slice 6 months after treatment (**B**) of recanalised PAVM showing successful occlusion with no persistence of PAVM. Pulmonary angiogram of recanalised PAVM (**C**) showing feeding artery (white arrow), venous sac (black arrowheads) and draining vein (black arrow) filling with contrast despite previous coil embolisation. Pulmonary angiogram (**D**) showing successful occlusion of PAVM with Amplatzer vascular plug (white arrow) and no filling of venous sac

**Fig. 2:**
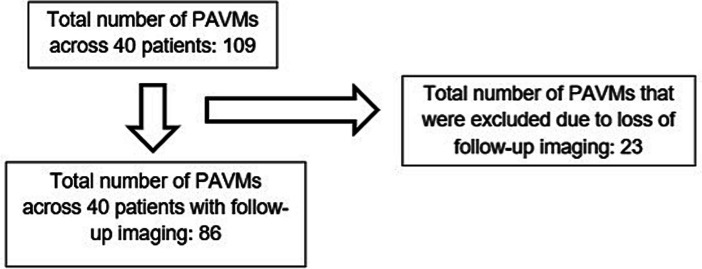
Flowchart of case inclusion and exclusion. The flowchart details the total number of patients and PAVMs identified, those meeting inclusion criteria, and those with available follow-up imaging. Reasons for exclusion from follow-up analysis are shown separately: incomplete clinical data, missing imaging, and technically inadequate imaging

In terms of embolisation agent, coil-only treatment demonstrated a non-significant trend toward higher recanalisation rates, while AVP and MVP use was associated with lower odds of recanalisation, though these did not reach statistical significance (Figs. 1 and 2, Table 2).

## Discussion

This study identified key risk factors associated with PAVM recanalisation following embolisation. On multivariable analysis, larger feeding artery diameter and complex PAVM morphology were the only independently significant predictors of recanalisation. Although male sex was associated with recanalisation on univariate analysis, this did not persist after multivariable adjustment and should be regarded as an exploratory finding. The results suggest that anatomical and morphological characteristics play a more prominent role than embolic device selection in determining treatment durability.

The observed association between larger feeding artery diameter and recanalisation aligns with existing literature. Pollak et al. [6] and Lee et al. [7] both reported that larger vessel size predisposes to incomplete occlusion or subsequent reperfusion due to increased flow and technical difficulty in achieving dense packing or complete sealing of the feeding artery. Larger arteries also pose challenges in achieving stable coil anchorage and are more likely to experience flow-related recanalisation, particularly when embolisation is performed proximally. Furthermore, larger sac size likely reflects a longer-standing or higher-flow lesion, which may increase the risk of incomplete thrombosis post-embolisation and predispose to reopening via residual microchannels or collateral recruitment.

The finding that complex PAVM morphology was independently associated with recanalisation underscores the importance of lesion architecture. Complex PAVMs, defined by multiple feeding arteries or draining veins, present technical challenges during embolisation. Occluding all feeding channels can be difficult, especially if some are small, tortuous, or difficult to catheterise. Previous studies [8, 9] have also highlighted that incomplete embolisation of complex PAVMs is a major cause of persistent shunting. This study’s findings therefore reinforce the need for pre-procedural cross-sectional imaging review to ensure that all feeding vessels are targeted during intervention.

The observation that male patients were more likely to experience recanalisation on univariable analysis is intriguing, though this did not persist in the multivariable model. Given the lesion-level analytical framework, patient-level variables such as sex were duplicated across multiple lesions from the same individual, which may artificially inflate apparent associations. This finding should therefore be considered hypothesis-generating rather than conclusive, and warrants further exploration in larger, multicentre cohorts to determine whether it represents a true biological effect or is related to unmeasured confounding factors such as lesion burden or flow dynamics.

Interestingly, the type of embolic material did not reach statistical significance, although a trend toward higher recanalisation rates with coil-only embolisation was observed. This trend is consistent with previous findings. Trerotola et al. [10] demonstrated that coils, especially when deployed proximally, may not provide durable occlusion due to the potential for recanalisation via residual flow channels between or around coil loops. Conversely, Amplatzer vascular plugs (AVPs) and Microvascular Plugs (MVPs) offer superior occlusion by providing a single-device, flow-occlusive mechanism that promotes immediate thrombosis and reduces the risk of migration. Although the lower recanalisation odds for MVPs did not reach statistical significance in the present study, this may reflect the relatively small sample size and corresponding wide confidence intervals. Recent studies [9–11] have suggested that plug-based embolisation achieves better long-term occlusion rates, especially for larger or complex PAVMs.

Notably, the presence of HHT did not significantly affect recanalisation risk in this cohort, contrasting with some prior reports. HHT-related PAVMs often demonstrate higher rates of persistence, potentially due to systemic angiogenic drive or multiple feeding channels [12]. The lack of significance here may be due to the small sample size or relatively homogeneous patient cohort, as 79% of participants had HHT, limiting statistical discrimination between groups.

## Limitations

This study has several important limitations that should be considered when interpreting the findings. First, the retrospective design limits causal inference and introduces the potential for selection bias. Second, follow-up imaging was available for 86 of 109 embolised PAVMs (78.9%), and the reasons for missing follow-up included loss to follow-up and technically inadequate imaging; this incomplete ascertainment may introduce bias and could lead to underestimation of recanalisation rates. Third, with a median follow-up of 12 months, this study primarily captures early to intermediate recanalisation. Late recanalisation, which is well described in the literature and may occur beyond 1 to 2 years post-procedure, is likely underrepresented, and true long-term recanalisation rates may be higher than reported. Fourth, analyses were conducted at the lesion level (86 PAVMs across 40 patients). Although cluster-robust standard errors were applied to account for within-patient correlation, this approach does not fully resolve non-independence introduced by including multiple lesions per patient. Patient-level variables such as sex and HHT status are duplicated across lesions from the same individual, which may artificially inflate associations. A formal patient-level sensitivity analysis was not feasible given the small sample size, and the association between male sex and recanalisation should be considered exploratory. Fifth, despite multivariable modeling, residual confounding cannot be excluded given the limited sample size and restricted number of covariates. Sixth, the recanalisation outcome was defined as less than 70% reduction in linear sac diameter, a pragmatic threshold based on prior literature rather than a universally accepted standard; variability in measurement methodology limits direct comparability with other studies. Finally, as a single-centre study, the generalisability of findings to other populations and institutional practices may be limited.

## Conclusion

The findings of this study demonstrate that larger feeding artery diameter and complex PAVM morphology are independently associated with an increased risk of recanalisation. Although specific procedural strategies and imaging techniques were not directly assessed, these results underscore the potential importance of careful procedural planning when managing high-risk lesions. Similarly, the data suggest that patients with larger or complex PAVMs may warrant closer post-procedure follow-up, although the optimal surveillance intervals remain to be established. Further studies are required to determine the most effective intervention techniques and follow-up protocols for reducing recanalisation risk.

## Data Availability

Available on request.
